# Assessing a New Prescreening Score for the Simplified Evaluation of the Clinical Quality and Relevance of eHealth Apps: Instrument Validation Study

**DOI:** 10.2196/39590

**Published:** 2022-07-05

**Authors:** Nicolas Wagneur, Patrick Callier, Jean-David Zeitoun, Denise Silber, Remi Sabatier, Fabrice Denis

**Affiliations:** 1 Institut Inter-régional Jean Bernard ELSAN Le Mans France; 2 Laboratoire de génétique chromosomique et moléculaire Centre Hospitalier Universitaire Dijon France; 3 Institut National de la e-Santé Le Mans France; 4 Centre d’Epidémiologie Clinique Hôtel Dieu Hospital Assistance Publique-Hopitaux de Paris Paris France; 5 Basil Strategies Paris France; 6 Service de Cardiologie Centre Hospitalier Universitaire de Caen Caen France

**Keywords:** scoring, eHealth, clinical relevance, solution, digital solution, clinical validation, prescreening, eHealth app, medical digital solution, scoring tool, health app, information quality

## Abstract

**Background:**

In 2020, more than 250 eHealth solutions were added to app stores each day, or 90,000 in the year; however, the vast majority of these solutions have not undergone clinical validation, their quality is unknown, and the user does not know if they are effective and safe. We sought to develop a simple prescreening scoring method that would assess the quality and clinical relevance of each app. We designed this tool with 3 health care stakeholder groups in mind: eHealth solution designers seeking to evaluate a potential competitor or their own tool, investors considering a fundraising candidate, and a hospital clinician or IT department wishing to evaluate a current or potential eHealth solution.

**Objective:**

We built and tested a novel prescreening scoring tool (the Medical Digital Solution scoring tool). The tool, which consists of 26 questions that enable the quick assessment and comparison of the clinical relevance and quality of eHealth apps, was tested on 68 eHealth solutions.

**Methods:**

The Medical Digital Solution scoring tool is based on the 2021 evaluation criteria of the French National Health Authority, the 2022 European Society of Medical Oncology recommendations, and other provided scores. We built the scoring tool with patient association and eHealth experts and submitted it to eHealth app creators, who evaluated their apps via the web-based form in January 2022. After completing the evaluation criteria, their apps obtained an overall score and 4 categories of subscores. These criteria evaluated the type of solution and domain, the solution’s targeted population size, the level of clinical assessment, and information about the provider.

**Results:**

In total, 68 eHealth solutions were evaluated with the scoring tool. Oncology apps (22%, 20/90) and general health solutions (23%, 21/90) were the most represented. Of the 68 apps, 32 (47%) were involved in remote monitoring by health professionals. Regarding clinical outcomes, 5% (9/169) of the apps assessed overall survival. Randomized studies had been conducted for 21% (23/110) of the apps to assess their benefit. Of the 68 providers, 38 (56%) declared the objective of obtaining reimbursement, and 7 (18%) out of the 38 solutions seeking reimbursement were assessed as having a high probability of reimbursement. The median global score was 11.2 (range 4.7-17.4) out of 20 and the distribution of the scores followed a normal distribution pattern (Shapiro-Wilk test: *P*=.33).

**Conclusions:**

This multidomain prescreening scoring tool is simple, fast, and can be deployed on a large scale to initiate an assessment of the clinical relevance and quality of a clinical eHealth app. This simple tool can help a decision-maker determine which aspects of the app require further analysis and improvement.

## Introduction

The number of eHealth tools has been expanding with the acceleration of innovation in telemedicine, connected objects, artificial intelligence, electronic patient-reported outcomes, immersive technologies, and other fields.

The COVID-19 pandemic further accelerated the emergence of new eHealth apps [1-3]. In 2020, 327,000 health apps were available on the Android and iOS App Store, and more than 250 eHealth solutions were added to app stores each day, or 90,000 in the year [4]. The number of health apps had doubled since 2013 [5].

However, there is great heterogeneity in the quality, relevance, and clinical performance of these solutions. It is difficult for users to differentiate the apps according to these 3 major criteria. It is also challenging for the providers of the eHealth apps to comply with good clinical practice. The technical developers may have no medical background or access to practicing clinicians. Most apps propose imprecise clinical benefits, and since they do not undergo any regulatory processes, their quality is uncertain and variable.

Whereas existing evaluation scores are often complex and difficult to deploy, health care institutions and the prescribers of these eHealth solutions need a simple, quick prescreening tool. However, there is no consensus on the benchmark for evaluating them in the context of clinical activity.

These prescreening tools must be based on good clinical practice guides and recommendations. Many standards and scoring methods already exist, and the first international recommendations for remote monitoring in oncology are now available [6].

A recent review of relevant medical literature analyzed the quality criteria for evaluating health solutions. Other criteria were then provided by the French National Health Authority (Haute Autorité de Santé; HAS), which is also responsible for the evaluation of drugs and medical devices [7,8]. Various other scores were also identified [9], such as mobile health evidence reporting and assessment [10], Digital Technology Assessment Criteria [11], ORCHA Review score [4], and MyHealthApps [12].

However, these scoring tools may include more than 150 questions, are laborious to use, and their effectiveness is yet unknown. Furthermore, they rarely evaluate all 4 key characteristics: clinical relevance, use potential, the quality of the provider, and the specificities of the solution.

We therefore set out to propose a rapid prescreening evaluation score. Although it can be used by any health care stakeholder, we determined 3 priority target users: eHealth solution designers, potential investors, and hospital decision-makers wishing to evaluate an existing or potential future solution. We developed the scoring tool to assess all aspects of eHealth good clinical practices and evaluated the key categories for 68 digital eHealth solutions.

## Methods

### Medical Digital Solution Scoring Tool

We built and made available the Medical Digital Solution (MDS) scoring tool, a new prescreening scoring tool based on 26 questions. We then used this tool to evaluate a panel of eHealth solutions [13].

The solution frontend was programmed with the ReactJS language. The application is hosted on a Hostinger server secured by an SSL protocol. The backend is based on the NoSQL Firebase solution. The technical functionality of the electronic questionnaire was tested by 10 editors before fielding the questionnaire. No cookies were used, and no IP check was done.

The design of the MDS scoring tool was based on the 2021 HAS Solution Evaluation Criteria [7], the HAS Good Practices Framework on Solutions and Connected Objects in Health (eHealth or mobile Health) of 2016 [14], and the European Society of Medical Oncology (ESMO) recommendations of 2022 [6].

This evaluation score was presented to the providers of eHealth solutions via a campaign on LinkedIn (a professional social network) from January 18, 2022, to January 30, 2022. The survey announcement is detailed in Multimedia Appendix 1, and the following is an English-translated excerpt:

How can we quickly assess the relevance and potential of a medical digital solution? We created the MDS trust score which aims to provide a rapid assessment of digital medical solutions tool for software publishers, patient associations, investors in the field of eHealth, and institutions. It is available to startups/solution publishers, associations, institutions, and investors...If you are interested in using it, please contact us.

The evaluation of the eHealth solutions was conducted via a close-access web solution URL [13]. The solutions retained for evaluation had to have clinical objectives. Wellness solutions were excluded, and we kept only the first evaluation to limit false score optimization biases. Only completed questionnaires were analyzed.

Solution providers were examined in light of the evaluation criteria and given a score in each of the 4 categories, as well as a total score. The categories included the specificities of the solution, the solution’s targeted population size and use potential, the clinical evaluation information of the solution, and provider information.

Part 1—solution specificities—evaluated the scope of the solution, the specialty concerned by the solution, the type of solution used, its compliance with the digital doctrine established by the HAS (a public agency reporting to the French Ministry of Health), the type of algorithm used by the solution, as well as its capacity of interacting with the user [15].

Part 2 assessed the solution’s target users based on age, user volume in France, the possibility for the use of the tool outside of France, the degree of its codevelopment with patients or patient associations, and the impact of the solution on the hospital organization.

Part 3 focused on the clinical evaluation of the solution, the outcomes used to assess the clinical benefit of the solution, the inclusion of feedback from medical specialists regarding the clinical relevance of the solution, the presence of support from or partnership with a scientific society, as well as the current level of clinical evidence of the solution.

Part 4 focused on the evaluation of the provider and included the presence of fundraising, the country of the headquarters, the presence of founding doctors on the board of directors, the presence of a medical department led by a physician, the presence and composition of a medical and scientific board, the media visibility of the solution on the internet, the development of previous eHealth solutions by the provider, and the presence or absence of a critical safety alert from the French National Agency for the Safety of Medicines. The strength and goals of the business model and reimbursement by French social security were also assessed. Among providers seeking reimbursement, we calculated a reimbursement probability score based on the clinical evaluation of the solution. The result was expressed in the semiquantitative form (low, medium, or high probability of reimbursement).

A score out of 500 was assigned to each of the 4 categories resulting in an overall score out of 2000, which was then reduced to a score out of 20. An example of the MDS tool is shown in Multimedia Appendix 2.

The questions and the weighting of the different answers to the 26 questions were designed by a group of 16 medical experts, eHealth experts, representatives of manufacturers and eHealth solution providers, methodologists, institutional evaluators, eHealth researchers, and representatives of patient associations.

A tool within the web platform also allowed providers to rank their solutions against other tested solutions.

### Ethical Considerations

No ethics review board assessment was required for this study of the characteristics of the solutions given the absence of patient data analysis and intervention. No demographic data were available, and their collection would not have been appropriate, as we only assessed solution characteristics and not the health data the solutions would collect.

### Statistical Analysis

We carried out a descriptive study of the characteristics of the solutions and assessed the scores of each solution by calculating the average, the median, and the first and third quartile distribution of the solutions. A Shapiro-Wilk test was performed to determine if the distribution of the score followed a normal distribution. For the chosen alpha level of .05, the scores were considered as normally distributed if the *P* value was >.05.

## Results

The MDS assessment score was used for 135 eHealth solutions via the web solution, and 68 solutions were assessable for our analysis. For the other solutions, the data were either incomplete (n=17) or duplicate (n=50). Incomplete forms were excluded from the analysis.

The 68 assessable solutions were associated with 102 clinical indications. Of the 102 clinical indications, 31 (30%) were related to support in taking medications, medicine compliance, and the reduction of treatment toxicity; 23 (23%) concerned the early detection of disease; 16 (16%) were related to decision support; 12 (12%) concerned prevention; 6 (6%) concerned direct therapeutic indications; 2 (2%) were related to patient triage; and 2 (2%) were aimed at relieving emergency department overload.

Of the 68 solutions, 22 (32%) targeted several medical specialties. Of the 90 specialties, the most present specialties were oncology with 20 (22%) solutions and cross-cutting solutions such as pain management with 21 (23%) solutions.

Of the 68 evaluable solutions, 29 (43%) were Class I medical devices according to the Medical Devices Directive 93/42/EEC of European Union [16] (Table 1).

Part 1 of the score concerned the study of general information about the solution (Table 2). Of the 68 evaluable solutions, 28 (41%) were based on nonartificial intelligence algorithms, and 22 (32%) were based on algorithms using artificial intelligence, of which 6 (9%) contained a nonintelligible artificial intelligence algorithm. We noted that almost all the algorithms (n=67, 99%) were less than 5 years old or otherwise up to date regarding the clinical standards within their specialty. Of the 68 solutions studied, 65 (96%) had a user interaction system and 32 (47%) were associated with remote monitoring with a health care professional.

**Table 1:** Solution characteristics (several items were possible per solution).

Characteristic			Solution, n (%)
**Solution area (2 items maximum per solution; N=102)**			
	Prevention	12 (12)	
	Early detection and flagging	23 (23)	
	Decision support	16 (16)	
	Treatment support, compliance, and toxicity reduction	31 (31)	
	Direct therapeutic solution (eg, virtual reality)	6 (6)	
	Patient triage	2 (2)	
	Emergency department decongestion	2 (2)	
	Others	6 (6)	
**Specialty concerned by the solution (2 items maximum per solution; N=90)**			
	Oncology	20 (22)	
	Cardiology	5 (6)	
	Neurology	6 (7)	
	Psychiatry	5 (6)	
	Pediatrics	2 (2)	
	Diabetology	7 (8)	
	Gynecology	5 (6)	
	Pulmonology	1 (1)	
	Nephrology	1 (1)	
	Urology and andrology	1 (1)	
	Rheumatology	2 (2)	
	Head and neck	1 (1)	
	Gastroenterology	0 (0)	
	Dermatology	3 (3)	
	Autoimmune disease, internal medicine, and infectiology	1 (1)	
	Surgery	3 (3)	
	Imaging	2 (2)	
	Geriatrics	3 (3)	
	Nutrition	1 (1)	
	Ophthalmology	0 (0)	
	Multiple specialties (emergency medicine, general medicine, and biology, etc)	21 (23)	
**Solution type (N=68)**			
	Nonmedical device (nonexecutive)	15 (22)	
	Nonmedical device (but theoretically should be)	10 (15)	
	Class I medical device	29 (43)	
	Class II medical device	12 (18)	
	Other CE^a^ markings (eg, Class III medical device)	2 (3)	

^a^CE: Conformitè Europëenne.

**Table 2:** Part 1: general information about the solutions.

Characteristic			Solution (N=68), n (%)
**Type of algorithm used in the solution**			
	No artificial intelligence (Boolean, common rules, and logistic regression, etc)	28 (41)	
	No algorithm	17 (25)	
	Intelligible artificial intelligence	16 (24)	
	Nonintelligible artificial intelligence	6 (9)	
	Algorithm more than 5 years old that were not reassessed with new support standards	1 (1)	
**Possibility of interaction with the user**			
	Yes, with remote monitoring with a health professional	32 (47)	
	Yes, alerts to the patient who then manages themselves	15 (22)	
	Yes, information not personalized according to the answers	10 (15)	
	Yes, patient alert with teleconsultation possible via the solution	3 (4)	
	Solution not affected	5 (7)	
	None, no user information	3 (4)	

Part 2 of the score concerned the study of the target population of the solution (Table 3). Of the 68 solutions evaluated, 42 (62%) covered several population age groups. The main age groups of the target populations were adults aged 18-64 years (58/110, 53% of age groups) and people aged ≥65 years (42/110, 38% of age groups). The median size of the population potentially reached by the solutions in France was 100,000 people. Of the 68 evaluable solutions, 59 (87%) were potentially applicable to the rest of the world. Patients had been involved in the solution development process in 59 (87%) solutions. The solutions tested typically facilitated the simplification of hospital organization (59%, n=40).

Part 3 of the score assessed the level of evidence and clinical relevance of the solution (Table 4). The clinical outcomes evaluated by the providers were heterogeneous and often multiple. In our study, we did not find a single, common criterion. Of the 169 validated outcomes, user satisfaction was cited 29 (17%) times, quality of life 24 (14%) times, medico-economic benefit 20 (12%) times, gain in early diagnosis 19 (11%) times, improved treatment compliance 19 (11%) times, and overall survival 9 (5%) times. Of the 68 solutions, 47 (69%) were assessed by experts as having major relevance, and 38 (56%) providers had benefited from the support of or partnership with a scientific society. Regarding the level of evidence of the solutions, 110 clinical evaluations were conducted for these solutions; 23 (21%) were randomized, 17 (15%) were prospective nonrandomized studies, 18 (16%) were retrospective studies, 28 (26%) were based on expert agreement, and 8 (7%) were not based on any studies or expert opinions.

**Table 3:** Part 2: the target populations of the solutions.

Characteristic			Solution, n (%)
**Age of targeted population (years; N=110)**			
	<18 (pediatrics)	10 (9)	
	18-64	58 (53)	
	>65	42 (38)	
**Number of patients involved in the codevelopment of the solution (N=68)**			
	>500	20 (29)	
	50-499	24 (35)	
	1-49	15 (22)	
	0	9 (13)	
**Impact on hospital organization (N=68)**			
	Simplification	40 (59)	
	Complication without associated act or package	7 (10)	
	Complication with associated act or package for financing the organization	4 (6)	
	No impact	17 (25)	

**Table 4:** Part 3: the clinical relevance of the solutions.

Characteristic			Solution, n (%)
**Validated outcome (several possible items per solution; N=169)**			
	User satisfaction	29 (17)	
	Quality of life	24 (14)	
	Medico-economic benefit	20 (12)	
	Early diagnosis gain	19 (11)	
	Improved access to care	19 (11)	
	Improved treatment compliance	18 (11)	
	Reduction of severity of a condition or symptom	13 (8)	
	Primary, secondary, or tertiary prevention	11 (7)	
	Survival	9 (5)	
	Reduction of emergencies	4 (2)	
	Less toxicity than reference	3 (2)	
**Level of evidence based on clinical assessment (several possible items per solution; N=110)**			
	Expert advice	28 (25)	
	Retrospective study of ≥300 evaluable patients	18 (16)	
	Applications of national or international recommendations in the solution	16 (15)	
	Randomized trial of <200 patients	13 (12)	
	Randomized trial of ≥200 patients or a meta-analysis	10 (9)	
	Prospective study of ≥200 patients versus nonrandomized control arm in real-life settings (historical comparison and data, etc)	11 (10)	
	Prospective study of ≥200 patients -) versus nonrandomized control arm not in real-life settings	6 (5)	
	Not based on any studies or expert opinions	8 (7)	

Part 4 the score concerned the characteristics of the provider (Table 5). Regarding provider fundraising, the answer was provided by 60 (88%) out of the 68 providers. Of these 60 providers, 30 (50%) had not yet raised funds, 11 (18%) had raised between €1.5 million (US$ 1.59 million) and €5 million (US $5.28 million), and 11 (18%) had raised >€5 million (>US $5.28 million). Of the 68 providers, 55 (81%) were based in France, 10 (15%) in other European countries, 1 (2%) in the United States, and 2 (3%) outside of Europe and the United States; 51 (75%) had a medical department that included at least one physician, of which 41 (60%) included at least one specialist in the field of the solution; and 60 (96%) had a scientific board with at least one doctor.

The media awareness of the solution over the past 12 months was assessed. On average, the providers or their solution were listed in 12 Google News search results, with a median of 7 search results. Of the 68 providers, 37 (54%) were developing their first eHealth app. Regarding the security of the solutions, 9 (13%) providers had had a security alert from the French National Agency for the Safety of Medicines. Of the 68 providers, 38 (56%) intended to obtain social security reimbursement for their solution. Of these 38 solutions, 26 (68%) had a low probability of reimbursement and 7 (18%) had a high probability.

**Figure 1:**
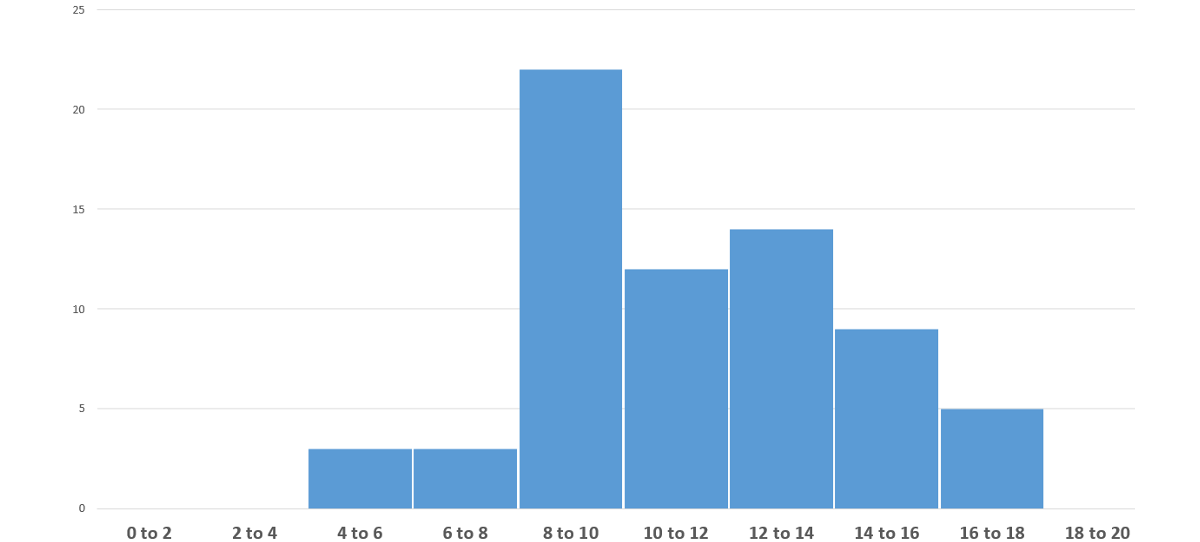
Overall rating distribution of the solutions according to overall score out of 20.

The calculation of the overall score is carried out for each eHealth solution by summing the points of the 4 previous criteria (Figure 1). The average score was 11.25 (range 4.7-17.4) points out of 20, the median score was 11.2 points out of 20, and the distribution followed a normal distribution (Shapiro-Wilk test: *P*=.33). The top 25% of apps scored below 9.4 out of 20, whereas the top 25% of apps scored above 13.4 out of 20.

**Table 5:** Part 4: information on the solution provider and reimbursement ambition and probability.

Characteristic			Solution, n (%)
**Presence of a medical director (N=68)**			
	Medical management by a specialist doctor	41 (60)	
	Medical management by a nonspecialist doctor	10 (15)	
	No medical direction	17 (25)	
**Development of previous eHealth solutions or medical device with >500 users (N=68)**			
	Yes, 3 or more	11 (16)	
	Yes, 2	7 (10)	
	Yes, 1	13 (19)	
	No	37 (54)	
**Business model (ambition or reimbursement as a goal; N=68)**			
	Yes (unless “device for collective hospital use”)	38 (56)	
	No, no need in the business model (sale of data and user subscription, etc)	23 (34)	
	No, device for collective hospital use	7 (10)	
**Probability of reimbursement (if an objective of the solution; n=38)**			
	High	7 (18)	
	Medium	5 (13)	
	Low	26 (68)	

## Discussion

### Principal Findings

We developed the first multidomain prescreening scoring tool to initiate an assessment of the clinical relevance and quality of a clinical eHealth app.

We proceeded to a first assessment of the relevance, quality, and level of evidence of an eHealth solution for 68 eHealth solutions available in France and reported the characteristics of the solutions in the different assessed fields.

The most represented medical fields were oncology (22%) and cross-cutting solutions covering several specialties (23%). This is confirmed by the literature; the specialty areas that have the most clinically validated eHealth solutions in terms of quality of life or survival are oncology and cardiology [17,18].

In our study, almost half (47%) of the evaluated solutions were based on a remote monitoring system deployed with a health professional. This type of solution occupies an important place in eHealth and is frequently used in cardiology and oncology. The HAS reported this as one of the most common configurations in its 2021 report evaluating solutions in the health sector [7]. The first international recommendations further stimulated the development of this type of instrument. These recommendations include the quality criteria to consider for the choice of these tools: the level of clinical evidence, the type of algorithm, Conformitè Europëenne marking, and the characteristics of the algorithms.

Evaluating the level of clinical evidence of a solution is an important step for the acceleration of its use in the medical world and possible reimbursement by health authorities. In 2022, this evaluation was a major criterion for obtaining a favorable recommendation from the ESMO for use [6]. We noted a great heterogeneity in the clinical evaluation criteria of the solutions. Only 5% of the solutions used overall survival as an endpoint. This is both one of the most difficult outcomes to obtain and the most relevant criterion according to the scientific community. Several remote monitoring solutions reduce mortality in patients followed for oncological or cardiac pathologies [19,20]. Survival is not an applicable outcome for the majority of the eHealth instruments. The criteria most frequently reported in our study are quality of life, gain in early diagnosis, better medico-economic benefit, or improved compliance with treatment. These criteria remain of interest in many solutions for the patients concerned.

In addition, in our study, 21% of the solutions were the subject of a randomized study. This type of study is considered as the highest degree of evidence and a major criterion to obtain reimbursement in France when undertaken. We also observed that 15% of the solutions had conducted prospective nonrandomized studies. The different types of study were weighted differently in our score. For example, conducting a randomized clinical study of ≥200 patients contributed 190 points out of 500 in the clinical evaluation score, whereas a retrospective study provided only 30 points.

About half (56%) of the providers declared the objective of obtaining reimbursement from French National Public Insurance, which in France covers the totality of the population by law. This possibility has been available in France since 2018. It adds a new business model modality to the development of a solution. We used the evaluation tool to identify the solutions that would have a high probability of reimbursement from the French National Social Security. This assessment was based on the type of studies conducted, as well as the type of clinical endpoints measured. In our study, 18% of the solutions among those seeking reimbursement were assessed as having a high probability of reimbursement.

Overall, the results of the evaluation of the 68 eHealth solutions seem close to the known elements of the literature. The average prescreening score of the evaluated solutions was 11.25 points out of 20. The scores ranged from 4.7 to 17.4. The distribution of the scores followed a normal distribution.

### Comparison to Prior Work

The first scores for evaluating eHealth solutions that appeared in the literature were mainly based on user or expert opinions [21]. The HAS listed 7 scores that focus on this scope (MyHealthApps [12], GGD Appstore [22], Health Navigator [23], One Mind [24], Osservatorio APP sanitarie [25], HealthOn [26], and the mobile app rating scale [27]). This type of evaluation is important in the development of a solution. In our study, 87% of the solutions involved patients in the development process, 75% had a medical department composed of at least one physician, and 96% had a scientific board with at least one doctor. These elements are important to optimize medical quality and therefore the trust and acceptability by patients of eHealth solutions.

The evaluation frameworks of other scores are typically descriptive, time-consuming, and qualitative tools to assess clinical quality. The Digital Technology Assessment Criteria designed by the National Health Service in the United Kingdom is an example of such an evaluation framework [11].

### Strengths and Limitations

We propose the calculation of a score based on our set of 26 evaluation criteria selected by a panel of experts and recommended in the literature. This score simultaneously evaluates the information on the solution, the target of the solution, the clinical evaluation of the solution, and the provider. This prescreening score has the advantage of being quick to achieve and having a wide range of evaluated criteria. The simple evaluation scores proposed in the literature often do not allow an evaluation that is as broad as our score’s [7]. Our questions are easily understandable and verifiable. This score can therefore be used by health care professionals including physician prescribers, pharmacists, and nurses; patient associations; investors; and providers to compare their solutions against competitors and track improvements of their solutions. The rapid realization of this score allows it to be regularly recalculated in real time for the same solution to improve the quality of the solutions.

Our score was assessed and validated in 68 eHealth solutions, unlike many other scores proposed in the literature that were not assessed in real life [7]. Notably, one of the most used scores today is the E-Solution Rating Scale [11].

Our prescreening score was developed based on recent and updated recommendations. It is based, among other things, on the recommendations of the HAS guides [7], as well as the recommendations of the ESMO released in 2022 [6]. However, the values of the different parameters will evolve according to new standards, recommendations, and data from the literature.

The limits of this score must also be taken into consideration. First, the score does not allow for an exhaustive and detailed assessment of all technical and clinical criteria. For example, the use of more detailed scores could be used to complete the assessment, such as the ORCHA Review score [28], which evaluates from 260 to 350 criteria; Enlight [29], which evaluates 476 criteria; and the framework from Henson et al [30], which evaluates 357 criteria. Second, the weighting of each answer was discussed by experts but empirically fixed in the absence of applicable quantitative benchmarks. Third, the filling in of the data was done directly by the solution’s providers in an autonomous and declarative way. This information was not verified. However, we excluded from the study providers who had not exhaustively filled in the entire questionnaire and duplicate providers when several questionnaires were completed for the same solution—always keeping only the first evaluation to limit false score optimization biases. Fourth, our tool does not conduct an in-depth assessment of the methodological quality of the reported studies. Other important characteristics of eHealth solutions are outside the scope of this score, including interoperability, security, portability, privacy, regulatory, ethics, and environment. Fifth, the short recruitment of the solution provider sample and the use of LinkedIn as a source can introduce a selection bias of the participants. For example, more sleep or mental health apps could have been assessed with a wider range of recruitment. Moreover, this recruitment led to only evaluating French solutions. Sixth, reimbursement processes are country-dependent, and the only geographical scope considered in our paper is France. The development of an international version of the score is in progress.

### Conclusion

We propose a multidomain prescreening tool that is simple and fast to use and usable on a large scale to initiate the evaluation of clinical digital solutions by any health care stakeholder. We believe that 3 target groups (eHealth solution designers, investors, and hospital decision-makers) will be the main initial users. This tool can help improve the quality of solutions and identify the aspects of the tools that may require further analysis and improvement. The score will be accessible on the website on the French National eHealth Institute [31] for the solution providers.

## Appendix

Multimedia Appendix 1 The published survey announcement.

Multimedia Appendix 2 Screenshot of the developed tool.

## Abbreviations

ESMO: European Society of Medical Oncology
HAS: Haute Autorité de Santé (French National Health Authority)
MDS: Medical Digital Solution

## Data Availability

The data sets generated during and/or analyzed during the current study are available from the corresponding author on reasonable request.

## References

[1] Galmiche S, Rahbe E, Fontanet A, Dinh A, Bénézit François, Lescure F, Denis F (2020). Implementation of a self-triage web application for suspected COVID-19 and its impact on emergency call centers: observational study. J Med Internet Res.

[2] Denis F, Galmiche S, Dinh A, Fontanet A, Scherpereel A, Benezit F, Lescure F (2020). Epidemiological observations on the association between anosmia and COVID-19 infection: analysis of data from a self-assessment web application. J Med Internet Res.

[3] Denis F, Septans AL, Periers L, Maillard JM, Legoff F, Gurden H, Moriniere S (2021). Olfactory training and visual stimulation assisted by a web application for patients with persistent olfactory dysfunction after SARS-CoV-2 infection: observational study. J Med Internet Res.

[4] ORCHA.

[5] Genève C (2020). État des lieux de la E-santé en 2020, étude d’une application mobile de santé [thesis]. Sciences pharmaceutiques.

[6] Di Maio M, Basch E, Denis F, Fallowfield L, Ganz P, Howell D, Kowalski C, Perrone F, Stover A, Sundaresan P, Warrington L, Zhang L, Apostolidis K, Freeman-Daily J, Ripamonti C, Santini D, ESMO Guidelines Committee (2022). The role of patient-reported outcome measures in the continuum of cancer clinical care: ESMO Clinical Practice Guideline. Ann Oncol.

[7] (2021). Évaluation des applications dans le champ de la santé mobile (mHealth): État des lieux et critères de qualité du contenu médical pour le référencement des services numériques dans l’espace numérique de santé et le bouquet de services professionnels. Haute Autorité de Santé.

[8] Riezebos RJ (2014). Peer-reviewing of mHealth applications: requirements for peer-reviewing mobile health applications and development of an online peer review tool [thesis]. University of Amsterdam.

[9] Azad-Khaneghah P, Neubauer N, Miguel Cruz A, Liu L (2021). Mobile health app usability and quality rating scales: a systematic review. Disabil Rehabil Assist Technol.

[10] Agarwal S, LeFevre AE, Lee J, L'Engle Kelly, Mehl G, Sinha C, Labrique A, WHO mHealth Technical Evidence Review Group (2016). Guidelines for reporting of health interventions using mobile phones: mobile health (mHealth) evidence reporting and assessment (mERA) checklist. BMJ.

[11] Digital Technology Assessment Criteria (DTAC). National Health Service.

[12] My Health Apps and blog. Patient View.

[13] (2022). Medical Digital Solution Score. Institut National de la e-Santé.

[14] (2016). Référentiel de bonnes pratiques sur les applications et les objets connectés en santé (mobile Health ou mHealth). Haute Autorité de Santé.

[15] (2022). La version 2021 de la doctrine du numérique en santé est en ligne !. Agence du Numérique en Santé.

[16] (1993). Council Directive 93/42/EEC of 14 June 1993 concerning medical devices. European Commission.

[17] Denis F, Krakowski I (2021). How should oncologists choose an electronic patient-reported outcome system for remote monitoring of patients with cancer?. J Med Internet Res.

[18] Georges JL, Cheggour S (2021). [Telemedicine, remote medicine, e-health:are French cardiologists ready?]. Ann Cardiol Angeiol (Paris).

[19] Denis F, Basch E, Septans A, Bennouna J, Urban T, Dueck AC, Letellier C (2019). Two-year survival comparing web-based symptom monitoring vs routine surveillance following treatment for lung cancer. JAMA.

[20] Lin Mao-Huan, Yuan Wo-Liang, Huang Tu-Cheng, Zhang Hai-Feng, Mai Jing-Ting, Wang Jing-Feng (2017). Clinical effectiveness of telemedicine for chronic heart failure: a systematic review and meta-analysis. J Investig Med.

[21] (2013). Niveau de preuve et gradation des recommandations de bonnes pratiques. Haute Autorité de Santé.

[22] GGD Appstore.

[23] Health Navigator.

[24] One Mind PsyberGuide.

[25] Osservatorio APP sanitarie.

[26] HealthOn.

[27] Stoyanov SR, Hides L, Kavanagh DJ, Zelenko O, Tjondronegoro D, Mani M (2015). Mobile app rating scale: a new tool for assessing the quality of health mobile apps. JMIR mHealth uHealth.

[28] Leigh Simon, Ouyang Jing, Mimnagh Chris (2017). Effective? engaging? secure? applying the ORCHA-24 framework to evaluate apps for chronic insomnia disorder. Evid Based Ment Health.

[29] Baumel A, Faber K, Mathur N, Kane JM, Muench F (2017). Enlight: a comprehensive quality and therapeutic potential evaluation tool for mobile and web-based eHealth interventions. J Med Internet Res.

[30] Henson P, David G, Albright K, Torous J (2019). Deriving a practical framework for the evaluation of health apps. Lancet Digit Health.

[31] Institut National de la e-Santé.

