# Supplementary figures and images for: Assessing a New Prescreening Score for the Simplified Evaluation of the Clinical Quality and Relevance of eHealth Apps: Instrument Validation Study
Source: J Med Internet Res. 2022 Jul 5;24(7):e39590. doi: 10.2196/39590 (PMC9297133; doi:10.2196/39590)

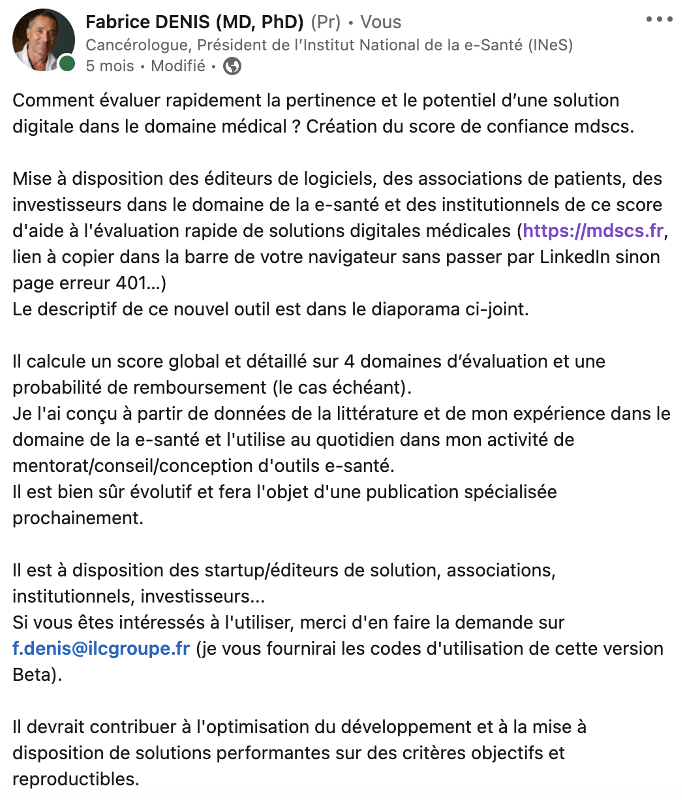

Supplement: Multimedia Appendix 1 [file jmir_v24i7e39590_app1.png]

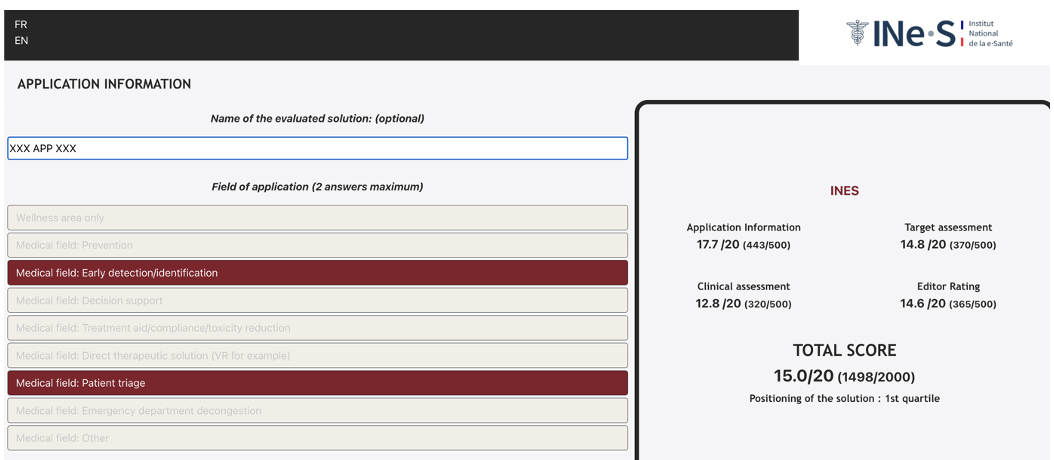

Supplement: Multimedia Appendix 2 [file jmir_v24i7e39590_app2.png]
